# Cranial irradiation disrupts homeostatic microglial dynamic behavior

**DOI:** 10.1186/s12974-024-03073-z

**Published:** 2024-04-03

**Authors:** Alexandra O. Strohm, Carl Johnston, Eric Hernady, Brian Marples, M. Kerry O’Banion, Ania K. Majewska

**Affiliations:** 1 Department of Environmental Medicine, University of Rochester Medical Center, Rochester, NY 14642 USA; 2 Department of Pediatrics, University of Rochester Medical Center, Rochester, NY 14642 USA; 3 Department of Radiation Oncology, University of Rochester Medical Center, Rochester, NY 14642 USA; 4 Department of Neuroscience, University of Rochester Medical Center, Rochester, NY 14642 USA; 5 Del Monte Institute for Neuroscience, University of Rochester Medical Center, Rochester, NY 14642 USA; 6 Center for Visual Science, University of Rochester Medical Center, Rochester, NY 14642 USA

**Keywords:** Radiation, Microglia, Two-photon microscopy

## Abstract

Cranial irradiation causes cognitive deficits that are in part mediated by microglia, the resident immune cells of the brain. Microglia are highly reactive, exhibiting changes in shape and morphology depending on the function they are performing. Additionally, microglia processes make dynamic, physical contacts with different components of their environment to monitor the functional state of the brain and promote plasticity. Though evidence suggests radiation perturbs homeostatic microglia functions, it is unknown how cranial irradiation impacts the dynamic behavior of microglia over time. Here, we paired in vivo two-photon microscopy with a transgenic mouse model that labels cortical microglia to follow these cells and determine how they change over time in cranial irradiated mice and their control littermates. We show that a single dose of 10 Gy cranial irradiation disrupts homeostatic cortical microglia dynamics during a 1-month time course. We found a lasting loss of microglial cells following cranial irradiation, coupled with a modest dysregulation of microglial soma displacement at earlier timepoints. The homogeneous distribution of microglia was maintained, suggesting microglia rearrange themselves to account for cell loss and maintain territorial organization following cranial irradiation. Furthermore, we found cranial irradiation reduced microglia coverage of the parenchyma and their surveillance capacity, without overtly changing morphology. Our results demonstrate that a single dose of radiation can induce changes in microglial behavior and function that could influence neurological health. These results set the foundation for future work examining how cranial irradiation impacts complex cellular dynamics in the brain which could contribute to the manifestation of cognitive deficits.

**Supplementary Information:**

The online version contains supplementary material available at 10.1186/s12974-024-03073-z.

## Introduction

Cranial irradiation (IR) is widely used in the treatment of various malignancies, with approximately 50–60% of cancer patients being treated with curative-intent or palliative radiation therapy [1–5]. In the United States alone, over 200,000 people undergo whole-brain irradiation (WBI) per year [6, 7]. Although effective, IR has long lasting consequences on human health. Up to 80% of patients experience symptoms of cognitive decline, including memory loss, motor dysfunction, and learning deficits that severely decrease their quality of life [8]. The mechanisms behind cognitive decline following IR remain poorly understood. Animal models are commonly used to examine how cellular changes may mediate cognitive effects following IR [9–15]. Growing evidence from these studies suggests that microglia, the highly motile resident immune cells of the central nervous system (CNS), contribute to the manifestation of cognitive deficits following IR [16]. Mice with depleted [9, 10, 13, 15, 17] or inhibited [14, 18, 19] microglia show reduced IR-associated neurological deficits. Precisely how microglia contribute to IR-induced cognitive decline is an active area of investigation and it is likely that complex changes and perturbations in homeostatic microglial behavior and function result from IR.

Microglia are highly reactive cells, undergoing changes in morphology that correspond with changes in function. IR has been shown to alter microglia morphology in several brain regions [20–22], suggesting that microglia deviate from their homeostatic functions in response to radiation. Studies in the hippocampus show a loss of neuronal structure and deficits in plasticity following IR [23] that are rescued by depleting [10, 13, 17] or inhibiting microglia [14, 18, 19, 24]. Hippocampal microglia isolated from irradiated mice show changes in gene expression indicative of classical reactivity [22, 25], further demonstrating that IR dysregulates microglia function in the hippocampus. In contrast, very little is known regarding how IR impacts cortical microglia. A number of neuronal effects have been reported in cortical areas following IR including increased neuronal excitation and injury [26], deficits in synaptic plasticity [27], and differential gene and neurotransmitter expression [28, 29]. Whether cortical microglial microglia are also sensitive to IR has yet to be explored.

Under homeostatic conditions, microglia are evenly distribution throughout the cortex and maintain distinct territories for efficient surveillance. Microglia constantly extend and retract their motile processes to monitor the functional state of the brain [30–32]. This probing of the environment allows microglia to perform vital functions in response to injury or during disease. Microglial processes also make dynamic, physical contacts with neuronal components to promote plasticity and structural remodeling [31–34], and changes in microglia process dynamics have been implicated in a variety of neurological diseases and disorders [35–40]. Microglia also undergo soma movement, exhibiting a modest level of displacement throughout the parenchyma under healthy conditions. This movement is regulated by changes in neuronal activity and can become irregular during disease or injury [30, 41–44]. The impact of IR on homeostatic microglial dynamics remains unclear.

Most studies to date have used static histological or transcriptional methods to assess microglial changes in response to irradiation, which limits our understanding of the temporal profile of microglial responses. Furthermore, assessing only a single time point in fixed tissue limits the ability to detect alterations in ongoing cellular dynamics within individuals over time. As a result, there is currently no information on whether IR disrupts the normal microglial dynamics required to maintain homeostasis. To understand the impact of IR on functions of these highly dynamic cells in a process that is temporally complex requires a dynamic in vivo approach. In this study, we paired in vivo two-photon microscopy with a transgenic model that labels cortical microglia to chronically follow these cells and determine how they change over time in irradiated mice and their control littermates. We exclusively studied male mice to minimize the number of animals utilized in our research, as extensive literature suggests that adult male mice exhibit greater sensitivity to the adverse cognitive effects of ionizing radiation compared to females [9, 10, 12, 24, 45–48].

## Results

**Fig. 1:**
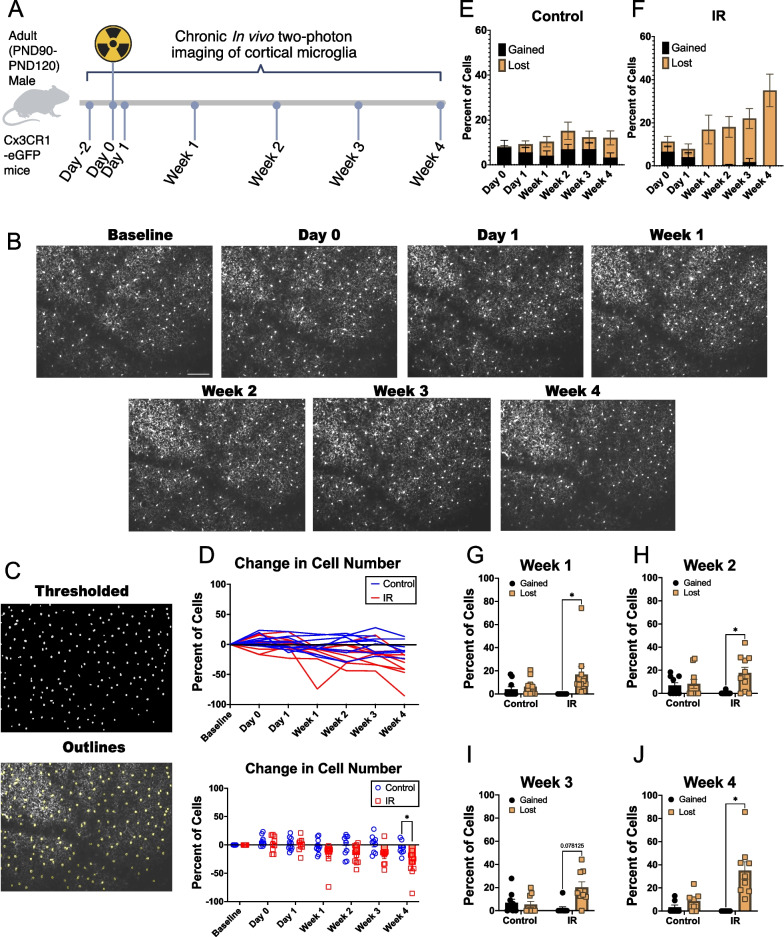
Microglia numbers decrease following cranial irradiation. **A** Experimental timeline (created with BioRender.com) **B** Representative images of microglia in the same area of the S1 over time at Baseline, Day 0 (5–9 h post-irradiation), Day 1, Week 1, Week 2, Week 3, and Week 4. Scale bar: 100 microns. **C** Example images of microglia after binarization (top) and outlines of microglia identified in binarized images superimposed on original images (bottom). **D** Percent change in microglial cell number for control and irradiated mice over time. Top panel shows individual animals over time while the bottom panel compares the percent change in cell number between control and IR groups. Percent of microglial cells lost or gained over time in **E** control and **F** Irradiated mice. Percent of microglial cells lost and gained at **G** Week 1, **H** Week 2, **I** Week 3, and **J** Week 4 in control and Irradiated mice. Mixed-effect analysis with Bonferroni’s post-hoc comparisons (**D**) or Wilcoxon matched-pairs signed rank test with Bonferroni-Dunn’s correction for multiple comparisons (**G**–**J**). * = p < 0.05. Data are presented as mean ± SEM. Each data point represents an individual animal. n = 7–10 mice per timepoint per group

To begin to assess the effects of IR on microglial behavior, we used adult male mice with microglia that express green fluorescent protein (GFP) under the control of the fractalkine receptor (CX3CR1^GFP/+^) promoter and exposed them to 10 Gray (Gy) cranial irradiation using the small animal radiation research platform (SARRP 225kVp X-rays). We previously showed this method is effective in delivering targeted irradiation with dosimetric precision to mice with cranial window implants [49]. We used only male mice for our study because adult male mice are reported to be more sensitive to cognitive effects following irradiation [9, 10, 12, 24, 45–48]. To track microglial properties and behavior over time, we imaged microglia in the same mice in the same area of layer 2/3 of the primary somatosensory cortex (S1) at different timepoints post-IR while under anesthesia. Blood vessels and stable cell somas were used to repeatedly relocate the same imaging area over time. We compared changes in microglial behavior and properties over time between irradiated mice and control littermates. We imaged cortical microglia two days prior to irradiation to account for any individual differences at baseline. We then irradiated (or sham irradiated) mice and imaged cortical microglia that same day (Day 0, 5–9 h post-irradiation) and the following day (Day 1) to assess any acute changes. To assess later changes, cortical microglia were imaged once every week following irradiation (Fig. 1A). We imaged cortical microglia over one month because previous studies have found cognitive deficits starting at four weeks post-irradiation [9–15], and we postulated that microglial behavioral changes would precede cognitive deficits.

### Cranial irradiation leads to microglia loss in the cortex

First, we wanted to determine whether IR could alter the microglial number over time in irradiated mice compared to control mice. We repeatedly imaged the same area of S1 up to four weeks post-irradiation to identify microglial somas (Fig. 1B, C). We measured the percent change in microglia cell number over time in irradiated mice compared to control mice normalized to the baseline, pre-radiation imaging session. We found reduced microglia cell numbers in the irradiated mice compared to the control mice starting at Week 1 which only reached statistical significance at Week 4 (Fig. 1D, mixed-effects analysis with Bonferroni’s post-hoc comparisons, p = 0.0431; Additional file 1: Fig. S1). When examining the percent of microglia cells lost and gained over time, control mice appeared to have a steady turnover with relatively balanced loss and gain of microglia over time (Fig. 1E), whereas the IR group had more cells lost than gained over time, especially after the first two days of imaging (Fig. 1F). When quantifying this, we found that at Week 1 through Week 4, irradiated mice had a greater number of lost cells than gained cells reaching statistical significance for weeks 1, 2, and 4 (Fig. 1G–J). This suggests that microglial cells are lost in S1 following IR, which is consistent with our previous findings in a pilot study [49].

### Microglia reorganize to compensate for cell loss following cranial irradiation

**Fig. 2:**
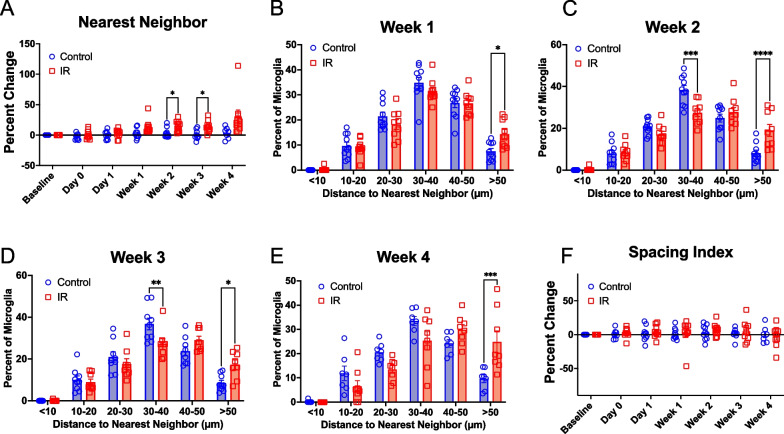
Microglia Reorganize to Compensate for Cell Loss Following Cranial Irradiation. **A** Percent change in the average nearest neighbor distance for microglia in control and Irradiated mice over time. Histograms showing the distribution of microglia nearest neighbor distances at **B** Week 1, **C** Week 2, **D** Week 3, and **E** Week 4 for control and irradiated mice. **F** Percent change in spacing index (squared average NND multiplied by the density for each image) for control and Irradiated mice over time. Mixed-effects analysis with Bonferroni’s post-hoc comparisons (**A**, **F**) or two-way ANOVA with Bonferroni’s post-hoc comparisons (**B–E**). * = p < 0.05, ** = p < 0.01. Data are presented as mean ± SEM. Each data point represents an individual animal. n = 7–10 mice per timepoint per group

Under homeostatic conditions, microglia are evenly distributed throughout the cortex. However, the ability of microglia to maintain territorial organization can be disrupted, resulting in altered spacing [38, 50–53]. We decided to examine whether IR impacted microglia distribution over a period of one month. We measured the nearest neighbor distance for each microglia to its neighbors within each timepoint for control and irradiated mice and normalized this to the baseline nearest neighbor distance to account for the local topography of the cortex. We found that at 2–3 weeks, irradiated mice had a significant increase in the average nearest neighbor distance between microglia, suggesting microglia cells were more widely dispersed compared to control mice (Fig. 2A, mixed-effects analysis with Bonferroni’s post-hoc comparisons, p = 0.0366 (week 2), p = 0.0311 (week 3)). This trend appears to begin at Week 1 and persists through Week 4, although it did not reach statistical significance at these two timepoints. To further assess differences in microglial territorial organization between groups, we examined the frequency distribution visualizing the percent of microglia whose nearest neighbors were at distances of < 10, 10–20, 20–30, 30–40, 40–50 or > 50 microns within each timepoint (Fig. 2B–E). We found there was a significantly increased percentage of microglia that were further away from each other in the irradiated group at more than a week after irradiation (Fig. 2B–E, greater than 50 microns away, Two-way ANOVA with Bonferroni’s post-hoc comparisons p = 0.0129 (week 1), p < 0.0001 (week 2), p = 0.0162 (week 3), p = 0.0002 (week 4)). This shows a shift in microglial cell distribution [50], suggesting microglia rearrange themselves to account for cell loss and maintain territorial organization following IR. It is possible that the microglia cells were further away from one another due to cell loss in the irradiated mice. Thus, we measured the spacing index, which accounts for changes in microglia cell density [50], finding no differences in the spacing index between control and irradiated mice (Fig. 2F), further supporting the notion that microglia redistribute to maintain territorial organization following IR.

### Cranial irradiation has subtle effects on cortical microglial displacement

**Fig. 3:**
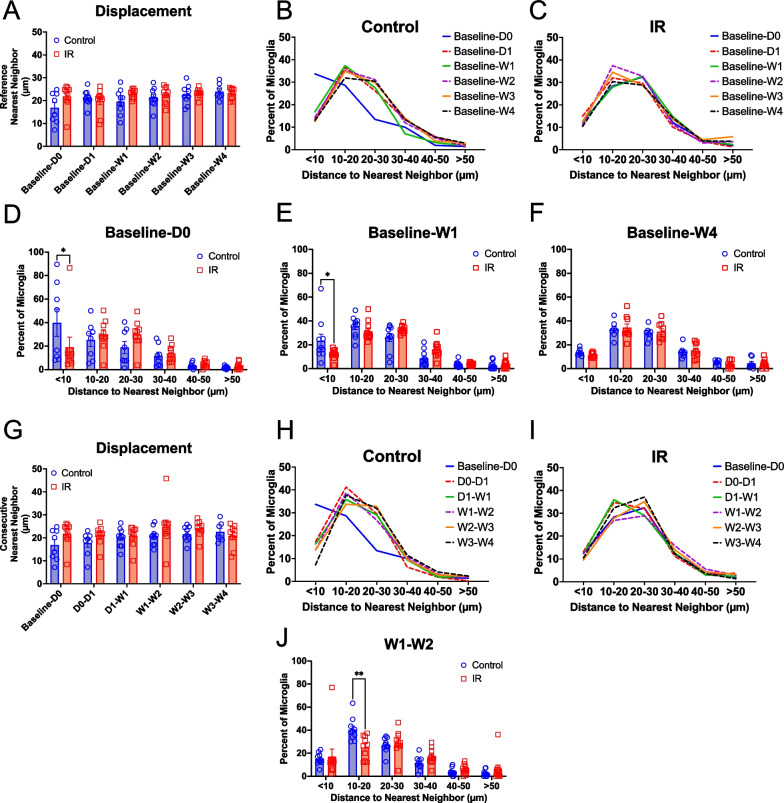
Cranial irradiation has subtle effects on microglial displacement in the cortex. **A** Displacement of microglial cells when comparing the baseline imaging session to subsequent timepoints in IR and control mice. The mean nearest neighbor distance did not change between IR and control mice. Histogram of displacement values for microglia comparing the baseline imaging sessions and subsequent timepoints in **B** control and **C** Irradiated mice. Comparisons of IR and control mice showed fewer stable microglia (with nearest neighbor distance of < 10 microns at **D** Day0, **E** Week 1, but not **F** Week 4 in IR mice. **G** Displacement of microglial cells when comparing consecutive imaging sessions in IR and control mice. The mean nearest neighbor distance did not change between IR and control mice. Histogram of displacement values for microglia comparing the baseline imaging session and subsequent timepoints in **H** control and **I** Irradiated mice. Notice that in this case, the consecutive comparison of the baseline imaging session to Day 0 is the same as in **A–D**. **J** Comparisons of IR and control mice showed fewer microglia with displacement of 10–20 microns between Week 2 and Week 3 only in IR mice. Mixed-effects analysis with Bonferroni’s post-hoc comparisons (**A** , **G**) or two-way ANOVA with Bonferroni’s post-hoc comparisons (**D–F**, **J**). * = p < 0.05, ** = p < 0.01. Data are presented as mean ± SEM. For panels **B**, **C**, **H**, and I SEMs were < 12%. Each data point represents an individual animal. n = 7–10 mice per timepoint per group for all panels

Although cortical microglia do not display large soma movements under healthy conditions, their displacement can increase during injury or disease [30, 41–44]. We examined whether IR dysregulates microglia movement over time by extracting microglial soma coordinates from each image and measuring the distance between each microglia and its nearest neighbor between different timepoints in irradiated mice and control littermates yielding a lower bound estimation of soma displacement over time. We first assessed microglial displacement by comparing each timepoint to the baseline imaging session as a reference (Fig. 3A–F). We found no differences in the average displacement of microglia in the irradiated group and the control group when comparing the baseline imaging session to subsequent timepoints (Fig. 3A). Most microglia in control and irradiated mice moved < 20 microns from their baseline location at all time points (Fig. 3B, C), which is similar to what has been previously reported under basal conditions [41, 42, 54]. In control mice, the histogram was skewed towards lower displacements when comparing baseline to Day 0, probably reflecting the proximity of the two imaging sessions in time. Interestingly, in irradiated mice this was not the case, suggesting that microglia may be particularly mobile right after irradiation. In fact, we observed fewer stable microglia (displacement < 10 microns) on Day 0 and Week 1 (Fig. 3D, E, Two-way ANOVA with Bonferroni’s post-hoc comparisons p = 0.0291, p = 0.0102). However, there were no differences in displacement when comparing baseline and Week 4 between the control and irradiated mice, with most microglia in both groups within 10–20 microns away from their baseline location (Fig. 3F). This may indicate that after one month, both control and irradiated microglia underwent similar amounts of movement from their location at the baseline imagining session.

Comparing nearest neighbor distances over imaging sessions that are separated by weeks to months can be prone to error if microglia undergo movements that distribute the population in similar patterns over time. This is because we are not tracking the movement of individual microglia, but rather the aggregate distributions of the population and thus cannot explicitly distinguish whether a microglial cell present in a similar location is the same microglia that was imaged previously. To partially account for this source of error, we compared displacement between consecutive timepoints (in this case most are one week apart) and found no differences in the average nearest neighbor distance between irradiated and control mice (Fig. 3G). In control mice, most microglia moved < 20 microns between consecutive timepoints (Fig. 3H), suggesting somas were relatively stable. In irradiated mice, starting a week after irradiation, most microglia moved between 20–30 microns between consecutive timepoints, which may suggest a higher mobility at later time points after irradiation (Fig. 3I). Despite this observation, when comparing the frequency distributions for displacement between consecutive timepoints, we found no significant differences between control and irradiated mice for most timepoints. However, between Week 1 and Week 2, irradiated mice had significantly fewer microglia that moved within 10–20 microns (Fig. 3J, Two-way ANOVA with Bonferroni’s post-hoc comparisons, p = 0.0024), again suggesting that irradiation may increase microglial mobility. Altogether, these results indicate that microglia in irradiated mice may be more mobile immediately following irradiation (Fig. 3D, Baseline-D0) and then again starting at Week 1 (Fig. 3E, Baseline to Week 1 and Fig. 3J, Week1 to Week 2). Thus, we observed a modest dysregulation of microglial displacement in the cortex within 2 weeks following IR, where generally fewer microglia in irradiated mice were stable compared to control mice.

### Cortical microglia morphology is unaffected by cranial irradiation

**Fig. 4:**
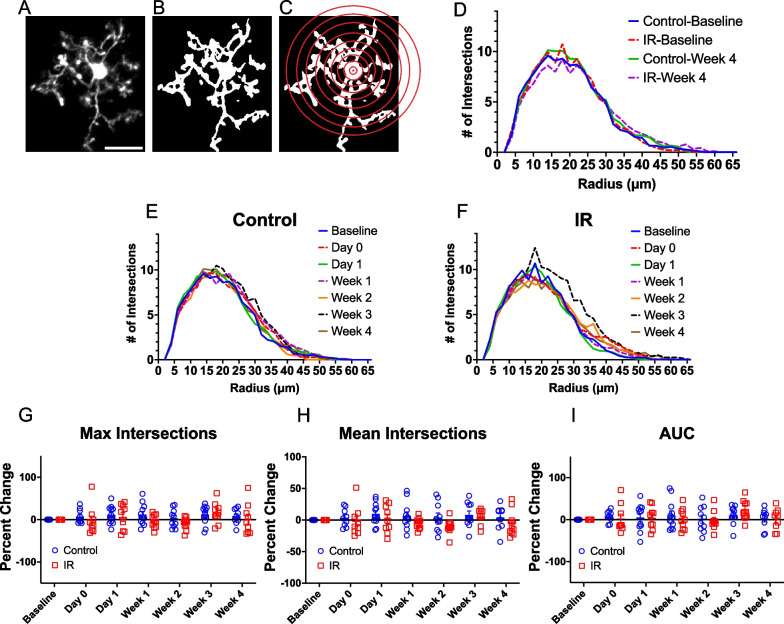
Microglia ramification is unaffected by cranial irradiation. **A** Example image of an individual microglia. Scale bar = 20 microns **B** Image of the same microglia after thresholding **C** Representation of Sholl analysis demonstrating concentric circles drawn at increasing radii from the center of the soma **D** Sholl curves for control and irradiated mice at baseline and Week 4. Sholl curves for **E** control and **F** irradiated mice at different time points. **G** Percent change in maximum number of intersections for control and irradiated mice over time. **H** Percent change in mean number of intersections for control and irradiated mice over time. **I** Percent change in area under the curve for control and irradiated mice over time. Mixed-effects analysis with Bonferroni’s post-hoc comparisons (**G–I**). Total cells analyzed per group: Control = 158, IR = 126. Data are presented as mean ± SEM. Each data point represents an individual animal. Sholl curves in **D–F** are presented as the mean for each group and timepoint specified. n = 8–11 mice per timepoint per group

Microglia are known to adopt an array of different morphologies that indicate functional changes in response to different stimuli [55, 56]. We examined individual microglia in the cortex and quantified morphological parameters to determine whether cortical microglia exhibit morphological changes following IR. First, we performed a Sholl analysis to assess microglial process ramification (Fig. 4A–C). We measured the number of intersections at increasing distances from the soma to generate Sholl curves for each timepoint following IR in irradiated and control mice (Fig. 4D–F). No differences were observed in the ramification of microglia between control and irradiated mice in the maximum number of intersections (Fig. 4G), mean number of intersections (Fig. 4H), or the area under the curve (Fig. 4I) at any timepoint examined.

**Fig. 5:**
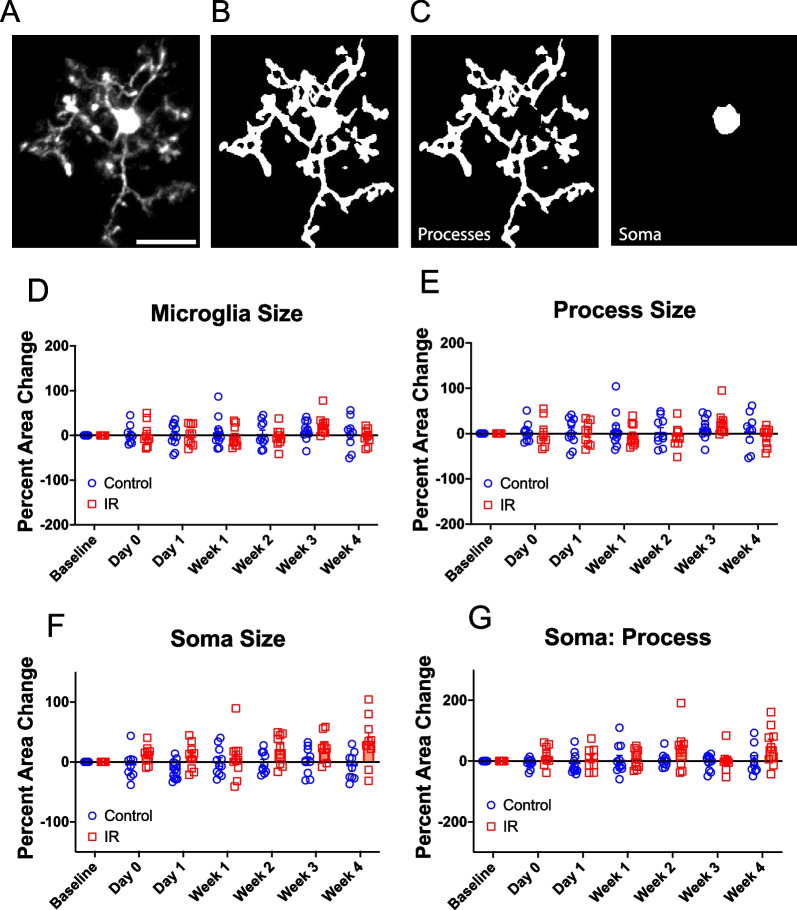
Microglia morphology is unaffected by cranial irradiation. **A** Example image of an individual microglia. Scale bar = 20 microns **B** Image of the same microglia after thresholding representing the whole microglial cell. **C** The microglial soma and processes were identified manually. **D** Percent change in microglia size for control and Irradiated mice over time. **E** Percent change in process size for control and irradiated mice over time **F** Percent change in soma size for control and irradiated mice over time **G** Percent change in soma to process ratio for control and irradiated mice over time Mixed-effects analysis with Bonferroni’s post-hoc comparisons (**D**–**G**). Total cells analyzed per group: Control = 158, IR = 126. Data are presented as mean ± SEM. Each data point represents an individual animal. n = 8–11 mice per timepoint per group.

We further assessed microglia morphology by examining microglia size and shape in irradiated and control mice. We measured the two-dimensional area (positive signal from binarized images) of microglial processes and somas to determine differences in size between control and irradiated mice (Fig. 5A–C). We found no differences in overall microglial cell size or process size between irradiated and control mice at any timepoint (Fig. 5D, E). Although there was an apparent increase in soma size between irradiated and control mice at several timepoints following irradiation, post-hoc testing indicated no statistically significant difference in soma size between groups at any timepoint (Fig. 5F). We also measured the soma to process ratio for each cell and found no significant differences between irradiated and control mice (Fig. 5G). Lastly, we assessed changes in microglia shape by measuring cell body and soma circularity, aspect ratio, roundness, and solidity (Additional file 2: Fig. S2A–H). We found no differences in any of these parameters between irradiated and control mice at any timepoint examined (Additional file 2: Fig. S2A–H), suggesting no effects of irradiation on microglial cell body or soma shape. Furthermore, principal component analysis of microglial morphology parameters revealed no clear separation based on radiation exposure (Additional file 3: Fig. S3A) or time (Additional file 3: Fig. S3B). Correlation analysis utilizing the Pearson correlation coefficient was executed on all combinations of morphology parameters to investigate the association between these parameters in both control and irradiated mice (Additional file 3: Fig. S3C&D). Comparable relationships were identified in most morphology parameters for both control and irradiated mice. Nevertheless, certain parameters exhibited distinct relationships; in control mice, soma size demonstrated a significant positive correlation with both process size and microglia size, whereas no significant correlation was observed in irradiated mice for these parameter pairs (Additional file 3: Fig. S3C&D). Notably, longitudinal studies of microglial morphology in the same animal over time have not previously been performed and our results suggest show little evidence of microglia undergoing time-specific morphological changes after IR in this region.

### Cranial irradiation reduces microglial coverage and surveillance capacity

**Fig. 6:**
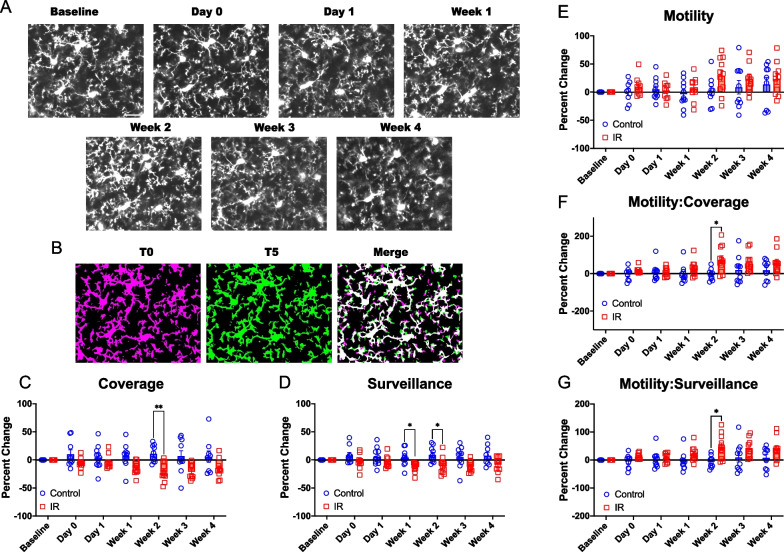
Microglia dynamics are altered following cranial irradiation. **A** Representative images used for dynamic measurements of microglia in the same area of cortex at different time points. Scale bar = 25 microns. **B** Representative image of binarized microglia at time = 0 min (T0) in magenta, time = 5 min (T5) in green, and merged timepoints. White represents pixels that are stable for both timepoints, while magenta represents retracted processes and green represents extended processes. **C** Percent change in microglial coverage for control and Irradiated mice over time. **D** Percent change in microglial surveillance index for control and irradiated mice over time. **E** Percent change in microglial motility Index for control and irradiated mice over time. **F** Percent change in microglial motility to coverage ratio for control and irradiated mice over time. **G**) Percent change in microglial motility to surveillance ratio for control and irradiated mice over time. Mixed-effects analysis with Bonferroni’s post-hoc comparisons (**C–G**). * = p < 0.05, ** = p < 0.01. Data are presented as mean ± SEM. Each data point represents an individual animal. n = 9–11 mice per timepoint per group

Microglia constantly survey their environment by extending and retracting their motile processes. Deviations from homeostatic levels of process dynamics are observed in animal models where neurological function is compromised [35–40]. We assessed whether homeostatic microglia process dynamics are disrupted by IR. Microglia were imaged at a 4X digital zoom to measure process dynamics by taking images every 5 min for 1 h (Fig. 6A, B). The area covered by microglia at T = 0 (first image) was used to assess microglial coverage (Fig. 6C). We found that microglial coverage appeared to decrease at early timepoints following IR compared to controls, and this decrease reached statistical significance at Week 2 post irradiation (Fig. 6C, Mixed-effects analysis with Bonferroni’s post-hoc comparisons, p = 0.0012), possibly reflecting the loss of microglial cells after irradiation (Fig. 1). We also quantified microglial surveillance by measuring the percentage of cortical area sampled by microglial processes over the hour-long imaging session, finding that irradiation decreased microglia surveillance, with statistically significant difference observed at Weeks 1 and 2 post irradiation (Fig. 6D, Mixed-effects analysis with Bonferroni’s post-hoc comparisons, p = 0.0346, p = 0.0366). This suggests microglia are occupying less of the parenchyma following IR, reducing their surveillance capacity. Next, we quantified microglial process motility by measuring microglial process extension and retraction. While microglial motility appeared elevated at later time points (Week 2–4) in irradiated mice, we did not find significant differences in the motility index between irradiated and control mice at any timepoint examined (Fig. 6E). We examined whether the relationship between motility and coverage or surveillance was impacted, as a reduction in coverage or surveillance could be compensated for by increased process motility. We therefore measured the ratio of microglial process motility to coverage as well as the ratio of microglial process motility to surveillance and observed an increase which reached statistical significance at week 2 (Fig. 6F, G, Mixed-effects analysis with Bonferroni’s post-hoc comparisons, p = 0.0275, p = 0.0157), but was elevated at Week 2–4 for both coverage and surveillance. This indicates a heightened level of microglial process motility relative to coverage or surveillance following IR. Principal component analysis of microglial dynamic parameters indicated a subtle shift in irradiated mice, without a distinct separation based on time (Additional file 4: Fig. S4A, B). Correlation analysis, employing the Pearson correlation coefficient, was conducted on all combinations of dynamic parameters to explore their associations in both control and irradiated mice (Additional file 4: Fig. S4C, D). Similar relationships were observed in most dynamic parameters for both groups, revealing a significant positive correlation between surveillance and coverage, as well as a significant negative correlation between coverage and motility in both irradiated and control mice (Additional file 4: Fig. S4C&D). Although not statistically significant, there seemed to be contrasting relationships between motility and surveillance for control and irradiated mice (Additional file 4: Fig. S4C, D). Further examination using simple linear regression demonstrated an inverse relationship between motility and coverage in both control and irradiated mice (Additional file 4: Fig. S4E, F).

## Discussion

**Fig. 7:**
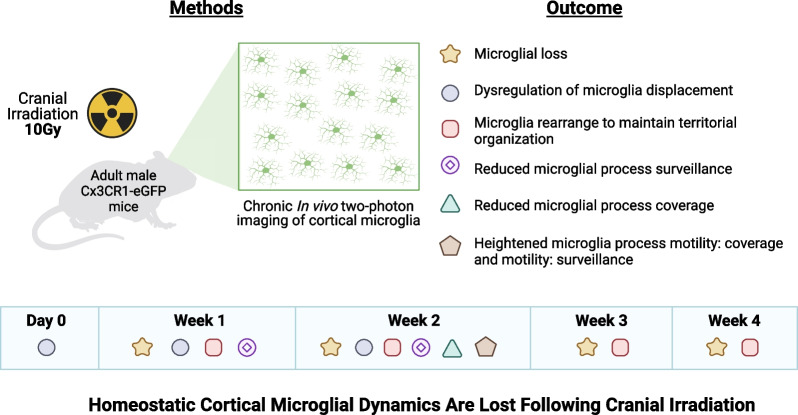
Summary schematic showing loss of homeostatic cortical dynamics at different timepoints following cranial irradiation. Created with BioRender.com

In this study, we report our novel findings on the effects of IR on cortical microglia dynamics (Fig. 7). The importance of microglia to the progression of IR-induced changes in the brain has been reported by several studies [9, 10, 13–19]. However, these studies largely concentrate on static snapshots of microglia at different times after IR. In contrast, microglia are highly dynamic cells that can rapidly change their morphologies and function, and they carry out their roles in the brain through their dynamic interactions with other cell types. To address the dynamic nature of microglia, we performed chronic in vivo imaging using two-photon microscopy to characterize cortical microglia number, morphology, and movement over one month following IR, allowing us to track the same areas of the brain over time to illuminate microglial dynamics on different time scales from minutes to weeks. We show a single dose of 10 Gy IR disrupts homeostatic cortical microglia dynamic behavior. We observed that IR resulted in microglial loss that persisted through one-month post-irradiation, and that microglia redistributed in the cortex, rearranging themselves to account for cell loss and maintain territorial organization. Furthermore, we found a modest dysregulation of microglial displacement in irradiated mice within 2 weeks following IR, indicating changes to microglial mobility. Lastly, we discovered that IR reduced microglia coverage and surveillance capacity, without overtly changing the morphology of individual microglia. These findings demonstrate that a single dose of IR can induce changes in microglial behavior and function, some of which persist over time and could contribute to the manifestation of cognitive deficits.

### Implications of microglia loss and irregular displacement on neurological health

We observed a ~ 30% loss of cortical microglia cells during the one month time course following IR, in line with previous studies that have shown microglial loss following IR both in the hippocampus [20, 57] and whole brain hemispheres [58]. IR may directly harm microglia, causing DNA damage and oxidative stress, leading to cell death and reactivity in surviving microglia. Alternatively, it could indirectly cause microglial injury by damaging nearby cells, triggering factors that result in microglial death and reactivity. Confirmation through tissue analysis using immunohistochemistry for cell death markers is needed to confirm this possibility. The loss of microglia could have strong negative consequences on neurological health and function. As the resident immune cells of the central nervous system, microglia are primarily responsible for defending the brain against pathogens and responding to injury. In a cortical stab wound injury model, IR can impair microglial proliferation and colony stimulating factor 1 receptor expression [59], demonstrating that IR can reduce microglial responses to injury. This impaired injury response coupled with a reduced overall microglial number could therefore render the brain more vulnerable to outside insults. Future experimentation using other secondary insults, such as a laser ablation time course, could further establish the relationship between radiation and diminished injury responses.

In the context of radiation injury, IR is believed to enhance microglial phagocytosis of synaptic elements, contributing to cognitive dysfunction [14, 16, 19, 24]. Depleting microglia in irradiated mice alters levels of synaptic proteins [13, 17], restores radiation-induced changes in spine morphology [13, 17], and rescues cognitive decline [9, 10, 13, 15, 17]. Given the substantial reduction in microglia population reported following IR, the remaining surviving microglia must exhibit strong functional changes to contribute to cognitive decline, despite their reduced numbers. Our study found that microglial distributions were shifted at weeks 1 through 4 following IR, indicating microglia rearrange themselves to account for cell loss and maintain territorial organization. We also observed a subtle effect of IR on cortical microglial displacement within 2 weeks following IR, where fewer microglia were stable in irradiated mice. Microglia soma movement can become irregular during seizures, altered sensory input, localized laser ablation, and in disease [42–44]. It is possible that microglia somas in irradiated mice migrate further away from their original locations at these earlier timepoints to respond to different injury signals in their surroundings or that they need to move to account for microglia loss which starts within a week in our experiments.

### Implications of reduced coverage and surveillance capacity on neurological health

Changes in microglia process dynamics can have serious implications for brain health and are observed in neurodegeneration, aging and neuroinflammatory models [35–40]. For example, microglia have reduced surveying capacity and response to injury with age [35, 36, 38]. In contrast, microglia can exhibit hypermotility that is also implicated in pathogenesis, as seen in mouse models of Alzheimer’s disease [40] and lipopolysaccharide-induced injury [39]. Although the increase in microglial motility that we observed following IR did not reach statistical significance, we did find a difference in process dynamics with reduced microglia coverage and surveillance observed following IR. A reduction in surveying capacity suggests less microglial contacts with their surroundings and an impaired ability to detect pathogens and damage signals. This coupled with the observed cell loss following radiation injury may leave the brain more vulnerable to secondary insults or injuries. Microglia also displayed a heightened process motility relative to coverage and relative to surveillance following irradiation. This could be a compensatory response, whereby microglia increase their process extension and retraction to counterbalance their reduced sampling area. However, relationships between motility and coverage were observed in both control and irradiated mice, suggesting that compensation in motility following IR may be part of a normal microglial compensation mechanism that exists during physiological conditions. Regardless of the mechanism, as microglia contact surrounding cells and synaptic components to engage in synaptic and structural plasticity [31, 32], their altered process dynamics in response to IR could therefore be reflective of their ability to engage in synaptic remodeling, which could lead to dysregulated synaptic phenotypes after IR [23, 24]. However, the implications of these disrupted dynamics on microglial interactions with their environment is speculative without further experimentation.

### Cranial irradiation effects on microglial morphologies

Microglia are highly heterogeneous, exhibiting an array of morphologies between and within brain regions that change depending on the function they are performing [56]. Generally, microglia with smaller somas and highly ramified processes are present in healthy adult mice. However, the functional meaning of different microglial morphologies is an area of active investigation. In models of neurodegeneration, aging, and injury, microglia exhibit changes in soma size, as well as process length and thickness [37, 38, 50, 60, 61]. Increases in microglia soma size and retraction of microglial processes could indicate several metabolic changes associated with classical microglia activation, oxidative stress, or increased lysosomal activity [60, 61]. In our study, we observed no changes in cortical microglial soma or process size, ramification, or shape between irradiated and control mice over time. Radiation induced-morphological effects differ depending on the sex, brain region, age at irradiation, timing of examination following irradiation, and type of irradiation used. Others have shown morphological changes in hippocampal microglia following IR [20, 22], which could mean that cortical microglia may be less susceptible to radiation effects than hippocampal microglia. However, the effects of IR on hippocampal microglial morphology at similar doses to our model (8–10 Gy) are reported to be transient, with most microglia resuming a ramified morphology within one day following irradiation [20, 22] and no differences observed between irradiated and control microglia by one month post-irradiation [22, 24]. Persistent changes in hippocampal microglial morphology have been observed at higher doses, which may be worth exploring in the cortex [62]. Although microglial morphology was unaffected in our study, there were functional differences in cortical microglial process dynamics, demonstrating cortical microglia are sensitive to radiation (Fig. 7). Our lack of detection of morphological differences could also be a consequence of small number of cells analyzed due to the limited imaging field of view required to capture fine microglial processes. It is important to note that while we imaged the same area in each animal over time, the microglia analyzed in this exact field of view may not be the same due to their mobility which may be particularly increased by irradiation (Fig. 3). Future studies could provide a more thorough analysis of regional differences in microglial morphological responses to radiation, as most studies have focused on hippocampal effects.

### Cortical radiation effects

Most of the radiation literature has focused on hippocampal effects as cognitive deficits are believed to be a result of loss of neuronal structure and impaired neurogenesis in this brain area [23, 63]. We examined the somatosensory cortex (S1) in our study as this area is ideal for chronic in vivo imaging [64] and because microglia play important roles in development, plasticity, and injury response of this area [65–67]. However, less is known about radiation effects in this brain region, although it is likely that sensory deficits contribute to cognitive decline post-radiation [68]. S1 receives peripheral sensory input from the thalamus and innervates the secondary somatosensory region that has links to the amygdala and hippocampus. S1 is responsible for sensory perception and identifies tactile characteristics, such as size, shape, texture and pain. This information is used for higher-order processing and problem-solving carried out by other brain areas. Though less is known regarding cortical radiation effects, there are reports of decreased cortical thickness and volume [69–71] and defects in sensory processing in patients following radiation treatment [68]. In rodent models, a number of radiation effects in cortical regions have been reported, including tissue necrosis [21], cellular senescence [72], changes in vascularization [21, 73], impaired neurovascular coupling [72, 74], astrocyte activation [21, 75], increased neuronal excitation and injury [26], and deficits in synaptic plasticity [27]. RNA sequencing studies on irradiated cortical tissue show differential expression of genes involved in circadian regulation, cell differentiation, and protein kinase activity [28]. Increased expression of excitatory neurotransmitters and receptors, coupled with increased glutamine/glutamate ratio has been observed, indicating a chemical imbalance in the cortex [29]. Altogether, these discoveries highlight the susceptibility of cortical areas to cranial radiation, which is further supported by the radiation effects on cortical microglial dynamics we report here. However, microglia are a heterogeneous population whose phenotypes and functions are tied to the brain area in which they reside [76], and therefore their contributions to radiation injury are likely brain region-dependent. Future studies examining multiple brain areas could help to uncover molecular mechanisms behind regional differences in radiation responses and how microglia contribute to these differences.

### Study limitations

It is important to note limitations to our study. First, our mice lack a functional copy of the fractalkine receptor, which could impact microglia radiation responses. Indeed, there is some evidence that fractalkine can regulate microglia radiation responses [77]. While these mice remain a gold standard for in vivo imaging, newly generated reporter lines which target different loci could be used to replicate these findings [78, 79]. Additionally, our Cx3Cr1 reporter transgenic line also labels peripheral macrophages. Others have sought to determine the extent of peripheral immune cell infiltration following cranial irradiation and found no evidence of infiltrating peripheral cells with doses of 5 or 8 Gy at these timepoints [20, 62, 80]. Furthermore, others report a single dose of 10 Gy does not affect the proportion [58] or result in the recruitment of peripheral macrophages [81], therefore we did not -attempt to distinguish resident microglia from infiltrating cells, although this should be examined in the future. We chose to examine only male mice because a large body of literature has shown adult male mice are more sensitive to the negative cognitive effects of IR compared to females [9, 10, 12, 24, 45–48]. However, it is possible that for the parameters we measured, female mice may also be affected by, or possibly even more sensitive to IR compared to males. Microglia structural and functional responses are indeed sex-dependent, with studies demonstrating microglia in male and female mice can have differential responses to insults [82, 83]. Though studies indicate males are more sensitive to IR in adulthood compared to females [9, 10, 12, 24, 45–48], female microglia can be more responsive to cranial irradiation during earlier developmental stages [84]. Sex differences should be examined in the future to determine whether IR impacts male and female microglial dynamics differentially. For our radiation model, we chose a single dose of 10 Gy, based on literature showing a single dose of 8–10 Gy results in cognitive deficits in mice starting at four-week post irradiation [9, 11–14]. It is possible that higher doses of radiation or a fractionated radiation scheme could have different effects on S1 microglia and this possibility should be explored. We also imaged and assessed microglial parameters over one month following irradiation, as we proposed that microglial behavioral changes would precede cognitive deficits. However, it is possible that microglia exhibit changes in dynamics after one month, as cognitive deficits [57, 85, 86] and immune responses [62, 80] have been reported at later timepoints following IR. While more technically challenging, cranial windows can be used to image the same cortical area for up to 6 months. Therefore, it may be beneficial in the future to study microglial dynamic behaviors at later timepoints following IR.

## Methods

### Experimental animals

Experiments were performed in accordance with the University of Rochester Committee on Animal Resources according to National Institutes of Health Guidelines. All procedures involving animals were approved by the Institutional Animal Care and Use Committee (IACUC) at the University of Rochester. CX3CR1^GFP/+^ mice in which microglia express green fluorescent protein (GFP) [87] were bred from crosses of CX3CR1^GFP/GFP^ and wild type C57BL/6 mice. All mice were exposed to 12 h of light and 12 h of dark and provided chow and water ad libitum. Only adult male mice were used in these experiments.

### Chronic cranial window preparation

Cranial window preparation was performed as described previously [88]. During surgery and imaging, mice were intraperitoneally administered an anesthesia cocktail containing fentanyl (0.05 mg/kg), midazolam (5.0 mg/kg), and dexmedetomidine (0.5 mg/kg). Mice were mounted on a stereotaxic frame and head fixed. Body temperature was maintained using a heating pad. A lubricating eye ointment was used to prevent eye drying. Aseptic technique was used throughout the surgery and tools were sterilized in between surgeries. The scalp was removed, and the skull exposed and cleared of debris and connective tissue. A 3-mm biopsy punch was used to mark the skull over the left S1, and a craniotomy was performed using a dental drill and a 0.5-mm drill bit (Fine Science Tools). A cranial window consisting of a circular 5-mm glass cover slip glued to a 3-mm glass coverslip (Warner Instruments) with UV glue (Norland Optical Adhesive) was placed with the 3 mm side down over the exposed dura. The window, surrounding skull, and incision site were sealed with C&B Metabond dental cement (Parkell) and a custom headplate (emachineshop.com; design courtesy of the Mriganka Sur lab, MIT) was attached. Slow-release buprenorphine (5 mg/kg, sc) was administered after surgery and mice were monitored for 72 h post-operation for signs of pain or discomfort. Mice were imaged at least two weeks after surgery to allow recovery from inflammation (~ 2 to 3 months of age).

### X-ray cranial irradiation

A small animal radiation research platform (SARRP) X-ray irradiator (225kVp XStrahl) was used to perform computed tomography (CT) image guided whole-brain radiation therapy as previously described [49]. Briefly, adult male mice (PND90-PND120) with cranial window implants were anesthetized with isoflurane and subjected a single 10 Gy dose from two parallel opposed beams using a 10X10 mm^2^ aperture. Irradiated mice were under anesthesia for less than 15 min. Nonirradiated control mice with cranial window implants were exposed to isoflurane, but were not placed in the SARRP irradiator.

### Two-photon imaging

A custom built two-photon laser scanning microscope (Ti:Sapphire laser, Mai-Tai, Spectra physics; Fluoview confocal scan head, BX61 microscope frame, 20 X 0.96 NA water-immersion objective, Olympus) was used to image layer 2/3 S1 cortical eGFP microglia in vivo. Excitation was achieved with 920 nm (100 fs pulse at 80 MHz) and emission was collected through a 580/180 bandpass filter, as described previously [49]. For chronic imaging of the same animal over time, the same area of the brain was identified for each imaging session and mouse by using the blood vessel and stable microglia as gross landmarks. Microglia were imaged under anesthesia at the following times for control and irradiated animals: Day 0 (5–9 h post-irradiation), Day 1 (24–36 h post-irradiation), Week 1 (6–7 days post-irradiation), Week 2 (13–15 days post irradiation), Week 3 (20–22 days post-irradiation) and Week 4 (27–29 days post-irradiation). Z-stacks were acquired with z-step size of 1 micron at 4X digital zoom (XY pixel distance of 0.25 mm) for morphology and dynamic analysis and at a 1X digital zoom for soma number, distribution, and displacement analysis. Image analysis was performed blind to treatment. Microglia parameters were analyzed only for mice with windows of sufficient quality for subsequent image analysis.

### Microglia soma number, distribution and displacement

Image data analysis was performed on a Macbook pro (2020) running MacOS 11.2. Regions of interest (ROI) were manually selected for areas that were present across timepoints. A principal components analysis was run on all raw images to reduce dimensionality of the image using a MatLab script. Z-stacks of 41 microns thickness were max projected and 20% of the dataset was used to train Ilastik software to recognize somas versus background, as previously described [54]. The resulting soma masks were thresholded and binarized in ImageJ/Fiji. The number of somas and XY coordinates for each soma were calculated using the “Analyze Particles” function in Fiji and extracted using a custom R script. For cell loss and gain measurements, the percent of cells that were lost (less than 0) or gained (greater than 0) relative to the baseline image were calculated for each animal at each timepoint. Gained and lost cells were matched for each mouse. The density was calculated as the total number of microglial cell somas divided by the ROI area. The 2D nearest neighbor distances (NND) were calculated within each timepoint to assess microglial soma distribution. The spacing index was calculated as the squared average NND multiplied by the density for each image [50, 54]. The frequency distribution microglial cells that had nearest neighbor distances of < 10, 10–20, 20–30, 30–40, 40–50 and > 50 microns was determined for each image. The 2D nearest neighbor distances between reference (baseline) and consecutive timepoints were used to measure microglial soma displacement. The frequency distribution of the percent of microglial cells that had nearest neighbor distances of < 10, 10–20, 20–30, 30–40, 40–50 and > 50 microns was determine between each timepoint for each animal. Each value was normalized to the baseline value (((value at timepoint/ value at baseline)-1)*100) to account for any variability at the start of the experiment and calculate the percent change in each parameter for each animal.

### Microglia process dynamics

Z-stacks of 51 microns thickness were acquired with a 1-micron step size at 4X digital zoom every 5 min for 1 h. Regions of interest encompassing 11 microns of tissue were manually selected for areas that were present across each timepoint in individual animals. Preprocessing in ImageJ/Fiji was performed using a custom script that performed 3D registration to correct for motion using the SIFT registration plugin, despeckling and Gaussian blur, and created maximum Z-projections for each timepoint and each animal as previously described [88]. Each image in the time series was analyzed separately, resulting in 12 images for each hour-long imaging session. Roughly 20% of the total dataset was used to train Ilastik software to recognize microglial cells versus background. The resulting microglia masks created were thresholded, binarized and recombined in ImageJ/Fiji to create xyt images (1 xyt image for each hour-long imaging session). Using MATLAB scripts previously created for this analysis as described in [88], binarized images of consecutive time points were overlaid. Positive pixels were classified as either stable (present in both time points), extensions (only present in second time point), or retractions (only present in first time point). The motility index was calculated as the sum of extended and retracted pixels divided by the stable pixels, averaged over the course of the imaging session. Surveillance is a measure of the total area surveyed by microglia during the hour-long imaging session. Surveillance was calculated by creating maximum T-projections from the binarized xyt image and determining the fraction of positive pixels. For analysis of microglial coverage, the first timepoint (T0) of each xyt image used for analysis. Coverage was calculated as the percent of the total area covered by positive binary pixels. For the motility to coverage ratio, the motility index for each animal and each timepoint was divided by the coverage. For the motility to surveillance ratio, the motility index for each animal and each timepoint was divided by the surveillance. All measurements were normalized to the baseline timepoint (((value at timepoint/ value at baseline) − 1)*100) to calculate the percent change for each animal.

### Microglial size, morphological and sholl analysis

The first timepoint (T0) for each xyt image was used for morphology analysis. Brightness/contrast, segmentation and cropping of individual microglia (one to four microglia per image per timepoint) were performed in ImageJ/Fiji. Roughly 20% of the total dataset was used to train Ilastik to recognize microglial cells versus background. The resulting microglial cell masks created were thresholded and binarized in ImageJ/Fiji and used for measurements of size, morphology and Sholl analysis. Somas were manually defined and cropped from each whole microglial cell for measurements of soma size and morphological parameters. Somas were removed from whole microglial cells to determine microglial process size. Size was calculated as the area of whole microglial cells, processes and somas using the “Measure” function. For the soma to process ratio, the soma size for each cell was divided by the process size. The “Analyze Particle” function was used to extract morphological data (circularity, area, roundness, solidity, and aspect ratio) from whole microglial cells and somas, described [54]. Circularity is equivalent to 4π (area/perimeter^2). For circularity, a value approaching 0.0 indicates an increasingly elongated shape, whereas a value of 1.0 indicates a perfect circle. The aspect ratio is the major axes/minor axes, which informs on the level of soma polarization. Roundness is the inverse of the aspect ratio, or 4*area/(π*major_axis^2). Solidity is the degree to which the area of the whole microglia or soma fills the area of its’ respective convex hull, which provides insights into the structural compactness and irregularity. Sholl analysis was performed on whole microglial cell images using the Sholl analysis plugin. Sholl curves were generated as the number of intersections versus the radius from the center of the cell soma. The maximum number of intersections, mean number of intersections, and area under the Sholl curve for each cell was calculated and extracted using a custom R script. All measurements were normalized to the baseline timepoint (((value at timepoint/ value at baseline) − 1)*100) to calculate the percent change for each animal.

### Statistical analysis

Sample size was estimated based on previous work using in vivo 2-photon microscopy where significant differences were detected in the parameters measured in this study [42, 89–91]. All statistical analysis was performed with GraphPad Prism version 9.5.1 for masOS, GraphPad Software, San Diego, California USA, www.graphpad.com or RStudio (Posit team (2023). RStudio: Integrated Development Environment for R. Posit Software, PBC, Boston, MA. URL http://www.posit.co/). For data comparing control to irradiated groups within a single timepoint, a Two-way ANOVA with Bonferroni’s post-hoc comparisons tests were performed. For comparing control to irradiated groups with data that was recorded from the same animal over time for multiple timepoints, a mixed-effects analysis (for data with missing time points for some animals) with Bonferroni’s post-hoc comparisons tests were performed. For Fig. 1G–J we used a Wilcoxon matched-pairs signed rank test with Bonferroni-Dunn’s correction for multiple comparisons (Fig. 1G–J), where gained and lost cells were matched for each mouse. PCA, correlation (creating a matrix with the Pearson correlation co-efficient and p-value), and linear regression analysis were performed in RStudio. A custom R script was used to unblind groups after analysis of different image parameters and make the graphs in supplemental Additional file 3: Fig. S3 and Additional file 4: Fig. S4.

### Supplementary Information

**Additional file 1: Fig. S1**. Changes inmicroglia numbers following cranial irradiation. Raw cell number changes in A) control and B) irradiated mice. Individual animals are represented by lines of different colors.

**Additional file 2: Fig. S2**. Cranial Irradiation does not change microglia or soma shape. Percent change in microglial A) circularity, B) aspect ratio, C) roundness, and D) solidity for control and irradiated mice over time. Percent change in microglial soma E) circularity, F) aspect ratio, G) roundness, and H) solidity for control and irradiated mice over time. Mixed-effects analysis with Bonferroni’s post-hoc comparisons. Data are presented as mean ± SEM. Each data point represents an individual animal. n = 8–11 mice per timepoint per group.

**Additional file 3: Fig. S3**. Characterization of Microglial Morphology Parameters in control and irradiated mice. Variation among morphology parameters color-coded by radiation exposure A) or time point B) revealed by PCA analysis. Morphology parameters analyzed included the following: Sholl max intersections, Sholl mean intersections, Sholl area under the curve, process size, soma size, microglia size, microglia circularity, microglial roundness, microglia solidity, soma circularity, soma aspect ratio, soma roundness, and soma solidity. Each data point represents an individual mouse at an individual timepoint. n = 8–11 mice per timepoint per group. Correlation matrix of the Pearson correlation coefficient of morphology parameters for control C) and irradiated mice D). Significant correlations (p < 0.05) are denoted with *.

**Additional file 4: Fig. S4**. Characterization of Microglial Dynamics in control and irradiated mice. Variation among microglial dynamics color-coded by radiation exposure A) or time point B) revealed by PCA analysis. Dynamics analyzed were surveillance, motility, and coverage. Each data point represents an individual mouse at an individual timepoint. n = 9–11 mice per timepoint per group. Correlation matrix of the Pearson correlation coefficient of microglial dynamics for control C) and irradiated mice D). Significant correlations (p < 0.05) are denoted with *. Simple linear regression between motility and coverage in control E) and irradiated F) mice.

## Data Availability

The data described in the paper is available upon reasonable request. Code used for image data analysis is available at https://github.com/majewska-lab.
