# Supplementary figures and images for: Cranial irradiation disrupts homeostatic microglial dynamic behavior
Source: J Neuroinflammation. 2024 Apr 3;21:82. doi: 10.1186/s12974-024-03073-z (PMC10993621; doi:10.1186/s12974-024-03073-z)

**A**

# Control

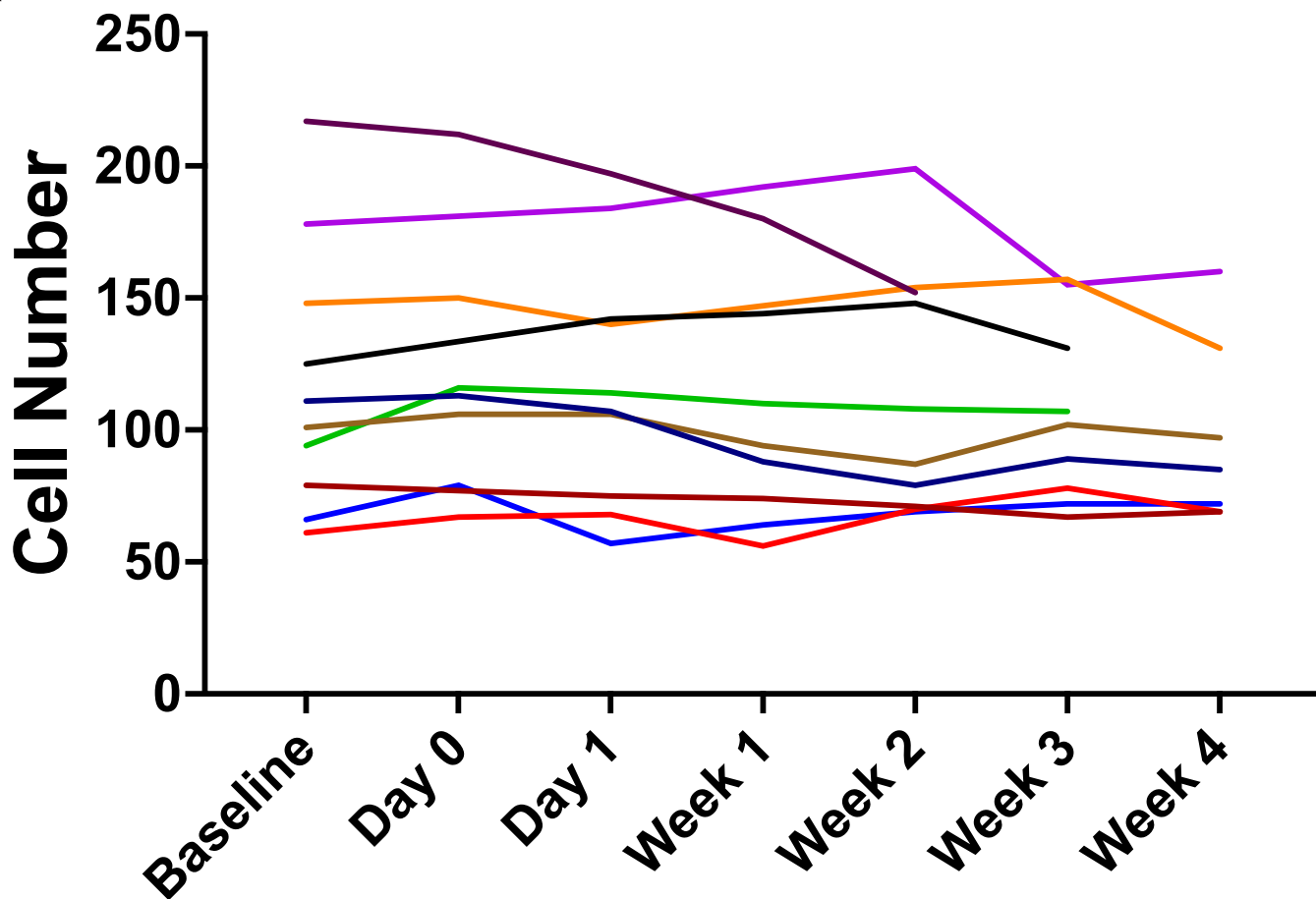**B**

# IR

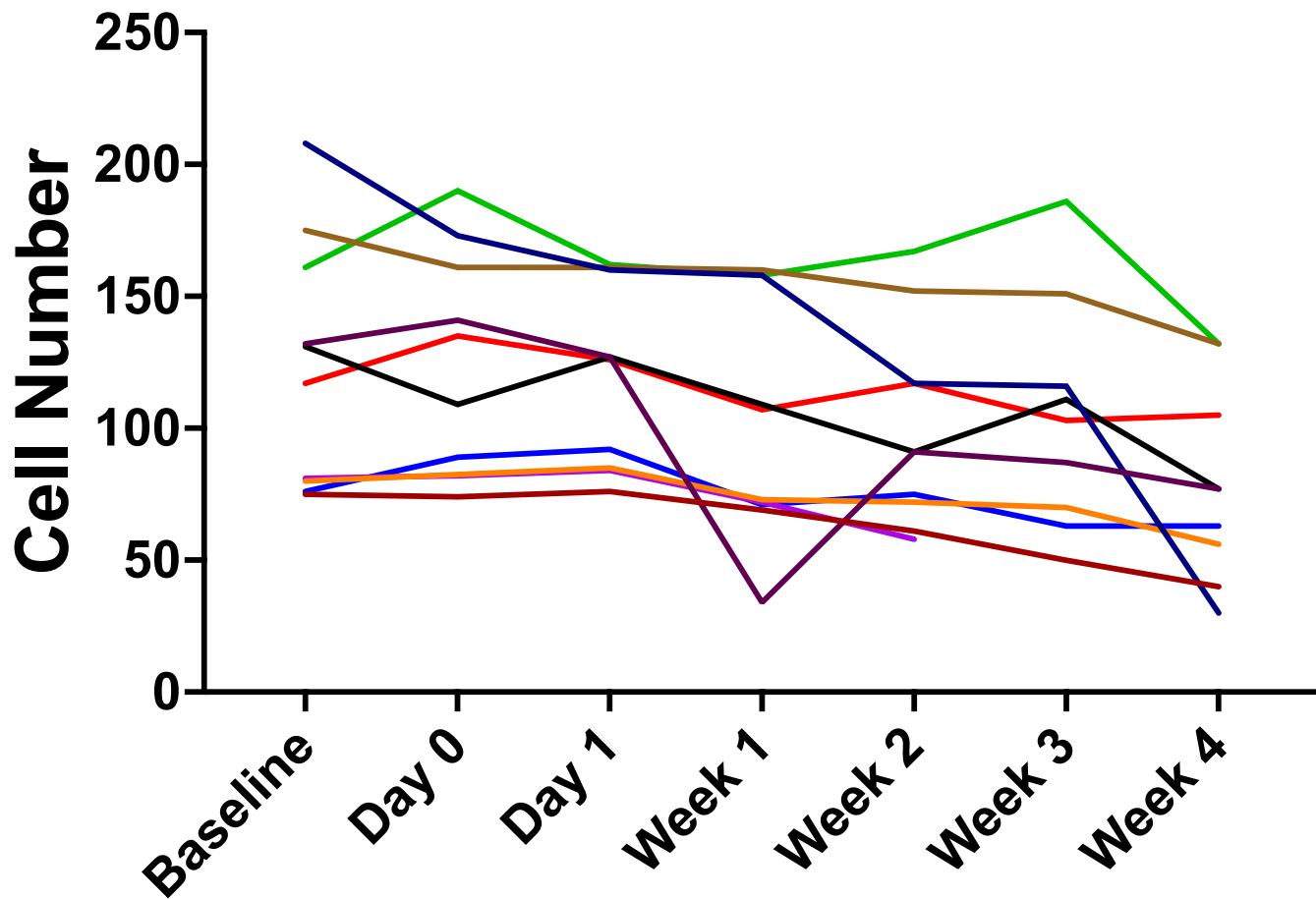

Supplement: Supplementary file 1 — Additional file 1: Fig. S1. Changes inmicroglia numbers following cranial irradiation. Raw cell number changes in A) control and B) irradiated mice. Individual animals are represented by lines of different colors. [file 12974_2024_3073_MOESM1_ESM.pdf]

# Microglia

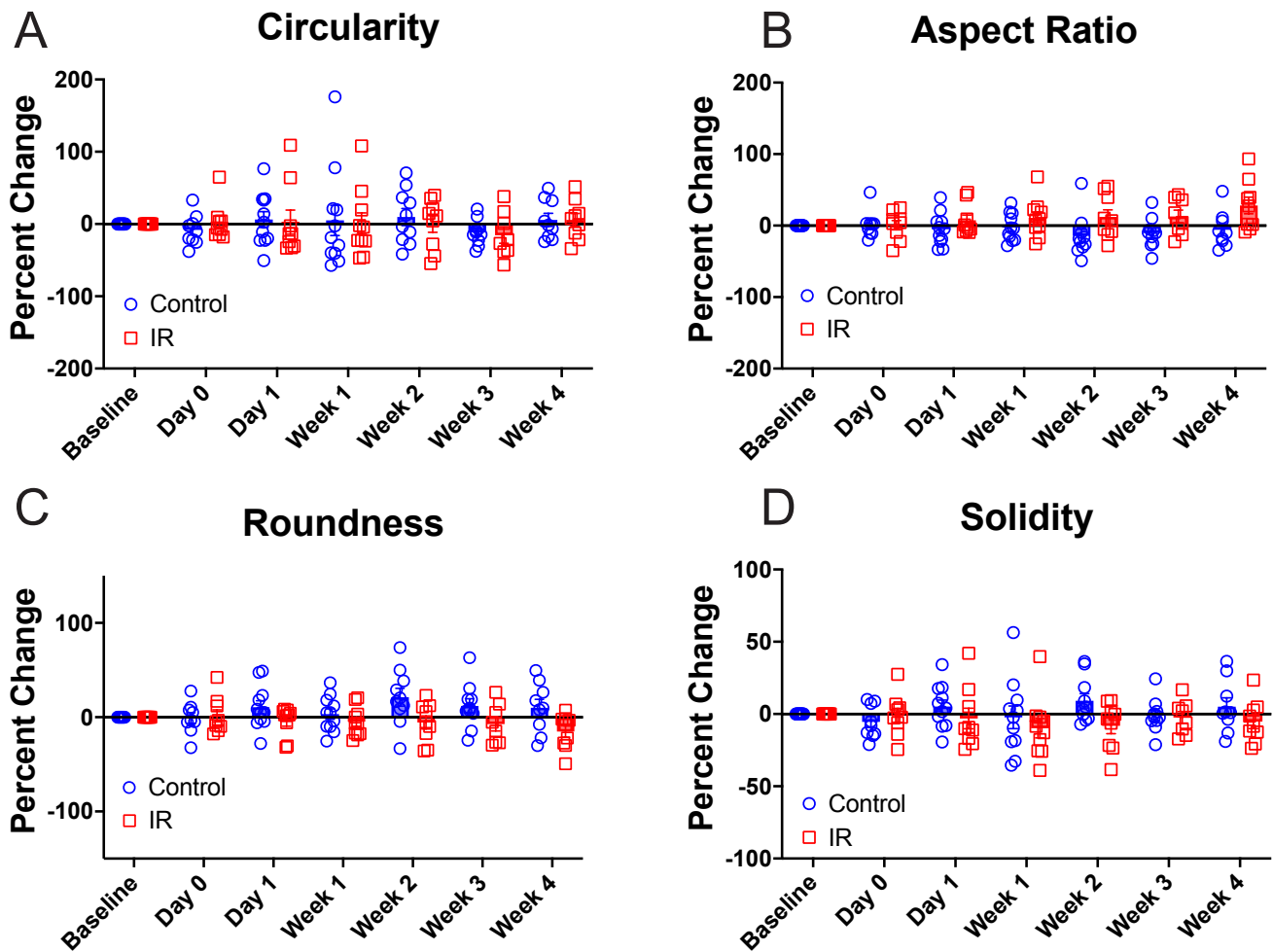

# Soma

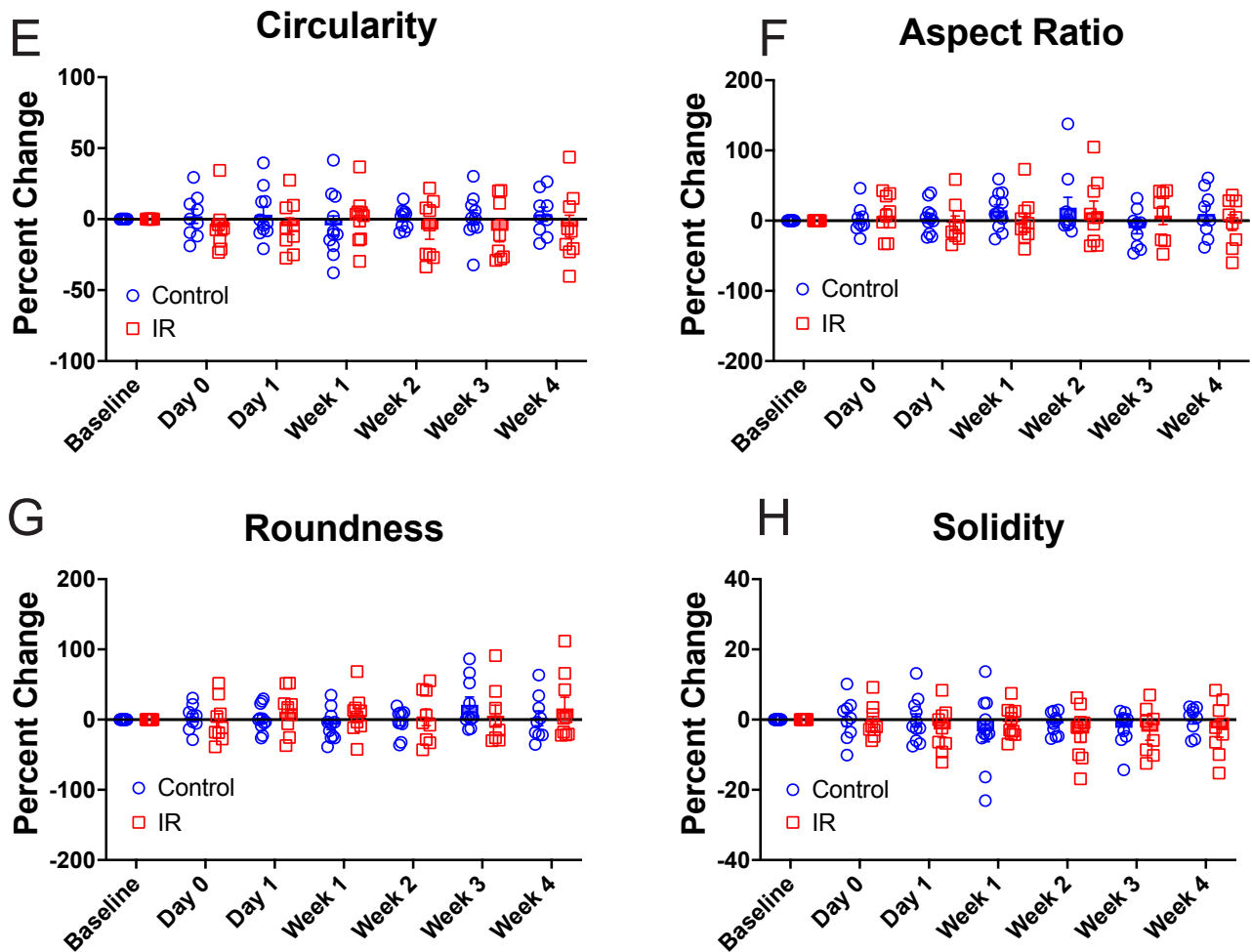

Supplement: Supplementary file 2 — Additional file 2: Fig. S2. Cranial Irradiation does not change microglia or soma shape. Percent change in microglial A) circularity, B) aspect ratio, C) roundness, and D) solidity for control and irradiated mice over time. Percent change in microglial soma E) circularity, F) aspect ratio, G) roundness, and H) solidity for control and irradiated mice over time. Mixed-effects analysis with Bonferroni’s post-hoc comparisons. Data are presented as mean ± SEM. Each data point represents an individual animal. n = 8–11 mice per timepoint per group. [file 12974_2024_3073_MOESM2_ESM.pdf]

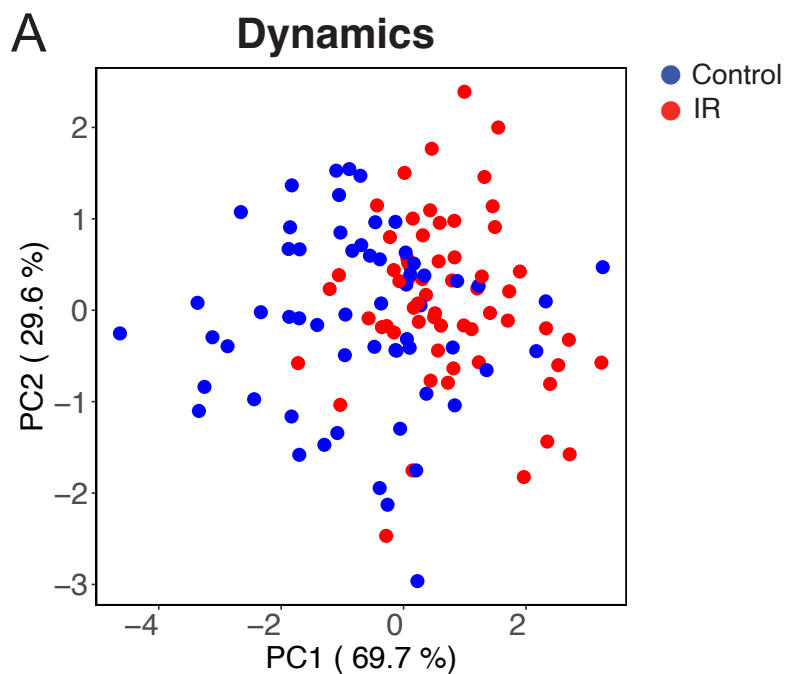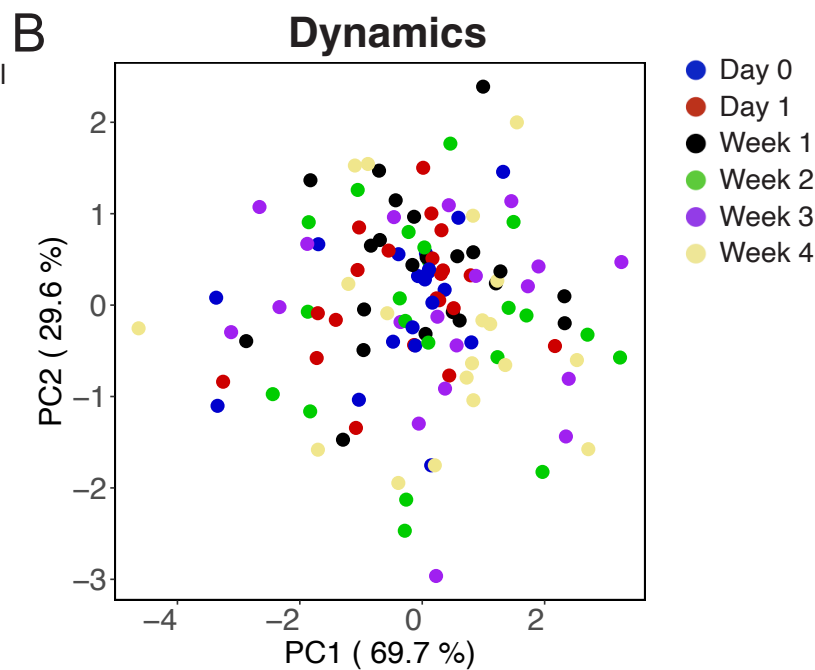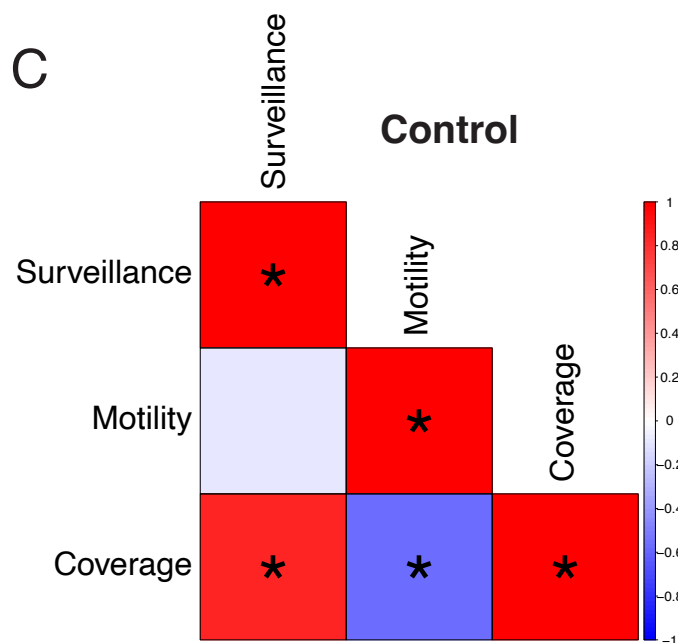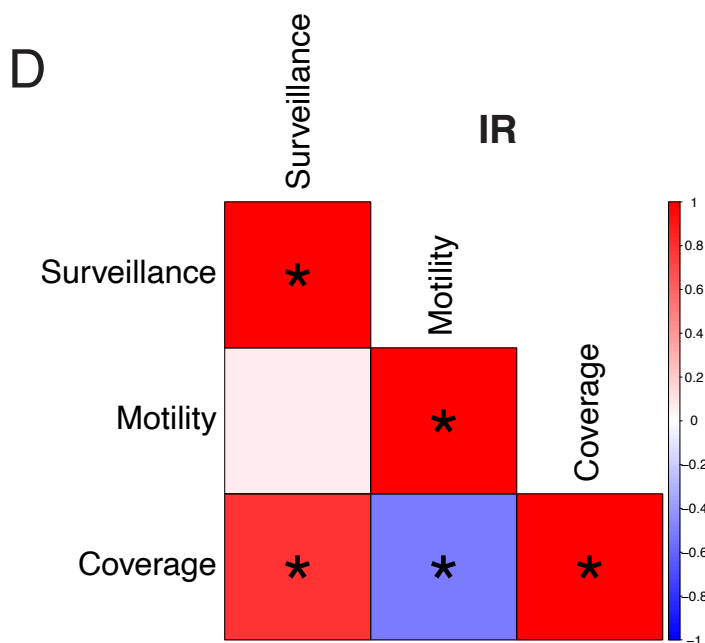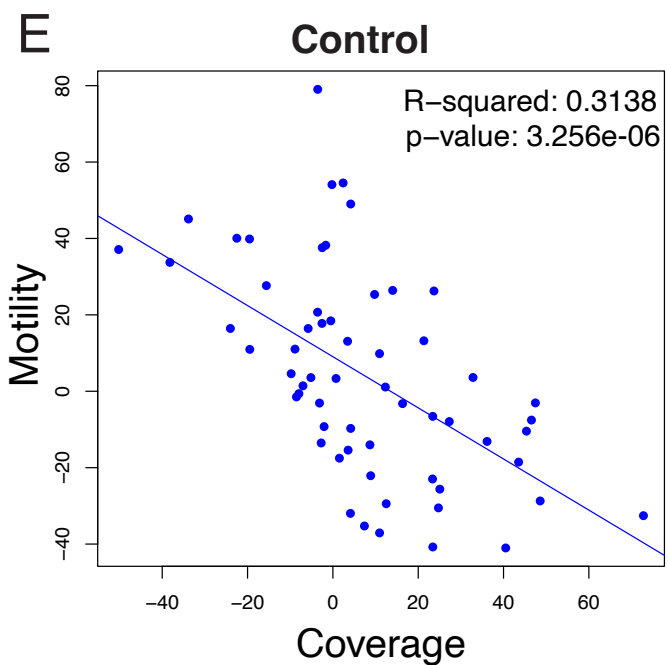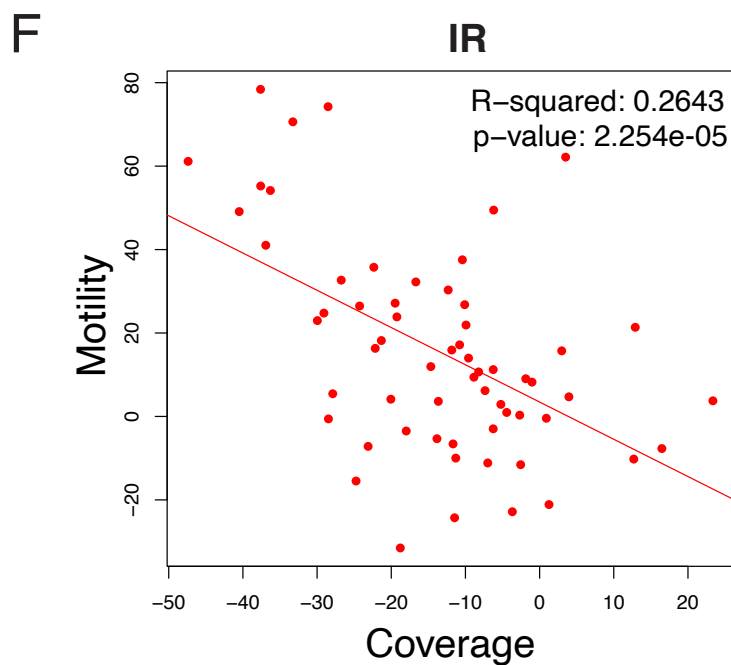

Supplement: Supplementary file 4 — Additional file 4: Fig. S4. Characterization of Microglial Dynamics in control and irradiated mice. Variation among microglial dynamics color-coded by radiation exposure A) or time point B) revealed by PCA analysis. Dynamics analyzed were surveillance, motility, and coverage. Each data point represents an individual mouse at an individual timepoint. n = 9–11 mice per timepoint per group. Correlation matrix of the Pearson correlation coefficient of microglial dynamics for control C) and irradiated mice D). Significant correlations (p < 0.05) are denoted with *. Simple linear regression between motility and coverage in control E) and irradiated F) mice. [file 12974_2024_3073_MOESM4_ESM.pdf]
