# Supplementary material for: Cranial irradiation disrupts homeostatic microglial dynamic behavior
Source: J Neuroinflammation. 2024 Apr 3;21:82. doi: 10.1186/s12974-024-03073-z (PMC10993621; doi:10.1186/s12974-024-03073-z)
Supplement: Supplementary file 3 — Additional file 3: Fig. S3. Characterization of Microglial Morphology Parameters in control and irradiated mice. Variation among morphology parameters color-coded by radiation exposure A) or time point B) revealed by PCA analysis. Morphology parameters analyzed included the following: Sholl max intersections, Sholl mean intersections, Sholl area under the curve, process size, soma size, microglia size, microglia circularity, microglial roundness, microglia solidity, soma circularity, soma aspect ratio, soma roundness, and soma solidity. Each data point represents an individual mouse at an individual timepoint. n = 8–11 mice per timepoint per group. Correlation matrix of the Pearson correlation coefficient of morphology parameters for control C) and irradiated mice D). Significant correlations (p < 0.05) are denoted with *. [file 12974_2024_3073_MOESM3_ESM.pdf]

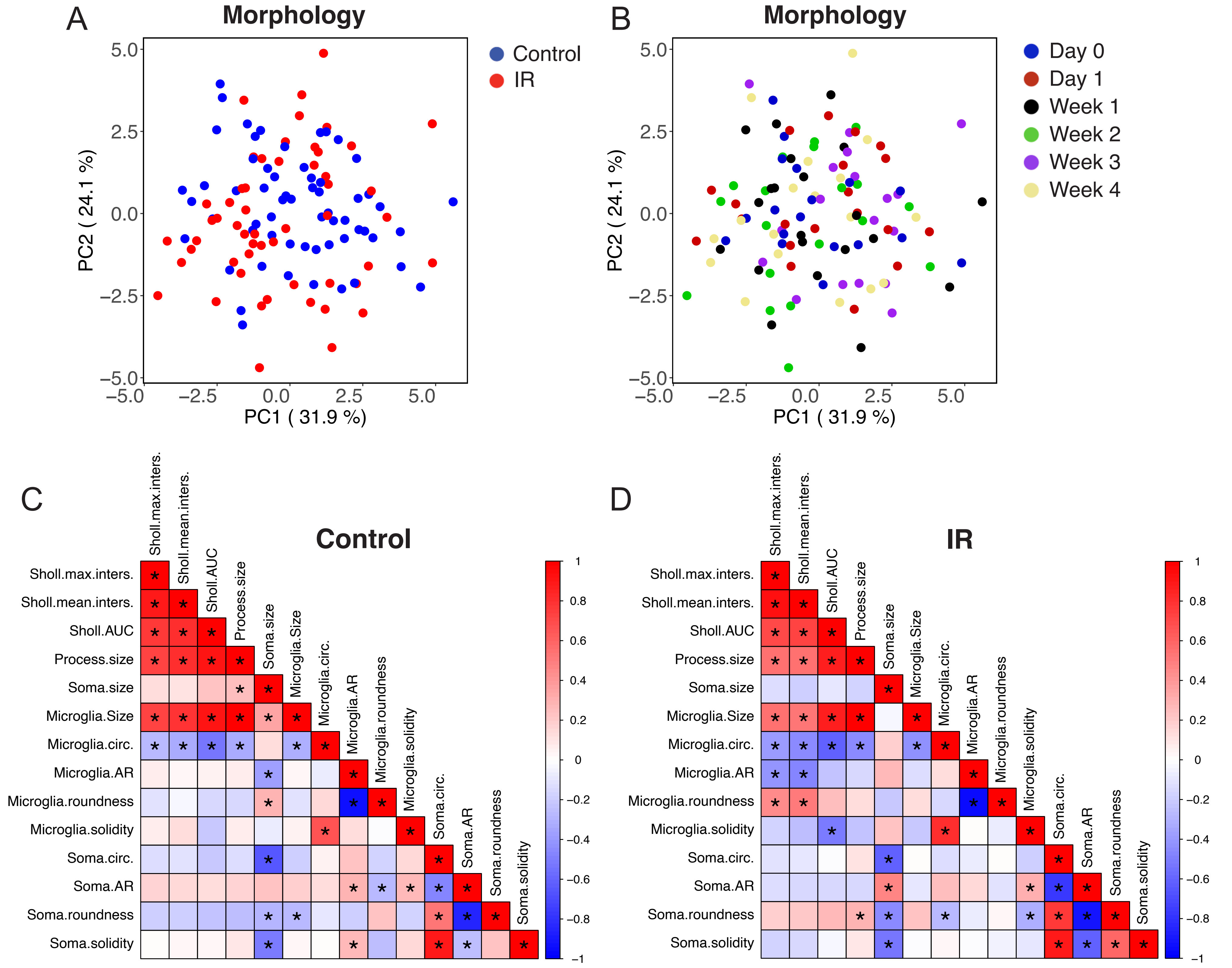
A
Morphology
Control
IR
PC1 (31.9 %)
PC2 (24.1 %)
5.0
2.5
0.0
−2.5
−5.0
B
Morphology
Day 0
Day 1
Week 1
Week 2
Week 3
Week 4
PC1 (31.9 %)
PC2 (24.1 %)
C
Control
D
IR
Sholl.max.inters.
Sholl.mean.inters.
Sholl.AUC
Process.size
Soma.size
Microglia.Size
Microglia.circ.
Microglia.AR
Microglia.roundness
Microglia.solidity
Soma.circ.
Soma.AR
Soma.roundness
Soma.solidity
1
0.8
0.6
0.4
0.2
0
−0.2
−0.4
−0.6
−0.8
−1
